# Role of 3-mercaptopyruvate sulfurtransferase in cancer: Molecular mechanisms and therapeutic perspectives

**DOI:** 10.1016/j.tranon.2025.102272

**Published:** 2025-01-14

**Authors:** Ka Zhang, Yi-Wen Zhu, Ao-Qi Tang, Ze-Tao Zhou, Yi-Lun Yang, Zi-Hui Liu, Yan Li, Xiao-Yi Liang, Zhi-Fen Feng, Jun Wang, Tong Jiang, Qi-Ying Jiang, Dong-Dong Wu

**Affiliations:** a Henan International Joint Laboratory for Nuclear Protein Regulation, School of Basic Medical Sciences, School of Stomatology, Henan University, Kaifeng, Henan 475004, China; b School of Clinical Medicine, Henan University, Kaifeng, Henan 475004, China; c Department of Stomatology, Huaihe Hospital of Henan University, School of Stomatology, Henan University, Kaifeng, Henan 475004, China; d School of Nursing and Health, Henan University, Kaifeng, Henan 475004, China

**Keywords:** 3-Mercaptopyruvate sulfurtransferase, Hydrogen sulfide, Polysulfide, Tumor, Tumorigenesis

## Abstract

The occurrence and development of tumor is mediated by a wide range of complex mechanisms. Subsequent to nitric oxide and carbon monoxide, hydrogen sulfide (H_2_S) holds the distinction of being the third identified gasotransmitter. Alternation of H_2_S level has been widely demonstrated to induce an array of disturbances in important cancer cell signaling pathways. As a result, the effects of H_2_S-catalyzing enzymes in cancers also attract widspread attention. 3-mercaptopyruvate sulfurtransferase (3-MST) is privileged to be one of them. In fact, 3-MST is overexpressed in many tumors including human colon cancer, lung adenocarcinoma, and bladder urothelial carcinoma. But it is also lowly expressed in hepatocellular carcinoma. In this review, we focus on the generation of endogenous H_2_S and polysulfides, facilitated by 3-MST. Additionally, we delve deeply into the potential role of 3-MST in tumorigenesis and development. The impact of 3-MST inhibition on the development of tumors and its potential for tumor therapy are also highlighted.

## Highlights

• 3-Mercaptopyruvate sulfurtransferase (3-MST) could produce hydrogen sulfide.
• 3-MST plays an important role in cancer development and progression.
• Regulation of the expression of 3-MST may have the potential for cancer therapy.
• More potent and selective 3-MST inhibitors need to be identified and developed.

## Graphical abstract

**Figure:**
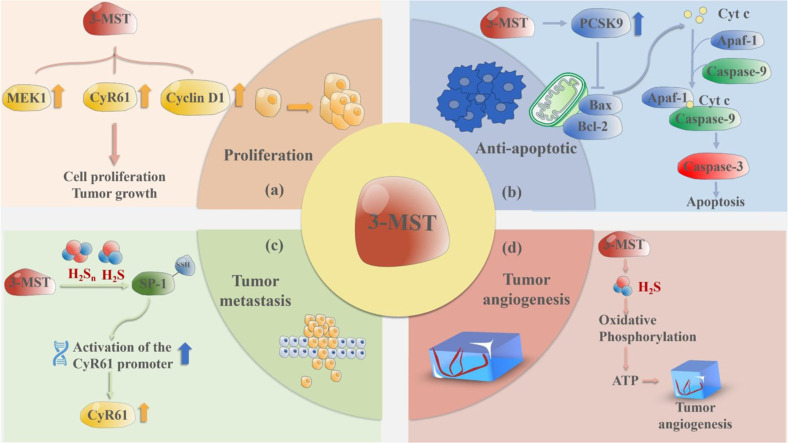

## Introduction

3-Mercaptopyruvate sulfurtransferase (3-MST) is a zinc-dependent hydrogen sulfide (H_2_S)-producing enzyme with a molecular weight of 33 kDa. The catalytic reaction can simultaneously generate polysulfides [1]. Unlike cystathionine beta-synthase (CBS) and cystathionine gamma-lyase (CSE), 3-MST does not depend on pyridoxal 5′-phosphate, and 3-mercaptopyruvate (3-MP) is the main substrate of 3-MST to produce endogenous H_2_S [2].

Recently, the physiological importance of 3-MST has gained the attention of researchers. 3-MST may have implications in a variety of disturbances, encompassing cardiovascular disease, neurological conditions, obesity, osteoarthritis, and liver damage, among others. The genetic deletion of 3-MST has a protective effect on cardiac ischemia-reperfusion injury (IRI) in 2–3 months old mice [3]. In spontaneously hypertensive rats, the expression of 3-MST in the heart is reduced, accompanied by a decrease in H_2_S [4]. Betaine can increase the levels of 3-MST, thereby preventing IRI in the rat brain [5]. A significant reduction in 3-MST activity is observed in the cortex and hippocampus of Alzheimer's disease mice, while the application with 3-MST substrate analog sulfanegen promotes the recovery of brain function [6]. Inhibition of 3-MST leads to the differentiation of preadipocytes into mature cells and increases lipid uptake, which may trigger obesity [7,8]. In summary, the disruption of 3-MST has been proven to be involved in the occurrence of many diseases.

The mortality rate of cancer ranks second globally [9]. According to authoritative statistics, the number of new cancer patients worldwide in 2018 reached 18.1 million cases, the number of people who lost their lives due to cancer is approximately 9.6 million [10]. The research on cancer therapies is growing exponentially, but currently there is still a lack of effective anti-cancer strategy. Exploring the underlying mechanisms of tumorigenesis and development will undoubtedly help identify cancer treatment targets. The expression of 3-MST has been discovered to increase in many tumors, indicating its important role in tumorigenesis and development.

## Previous studies on 3-MST

### The discovery of 3-MST

**Fig. 1:**
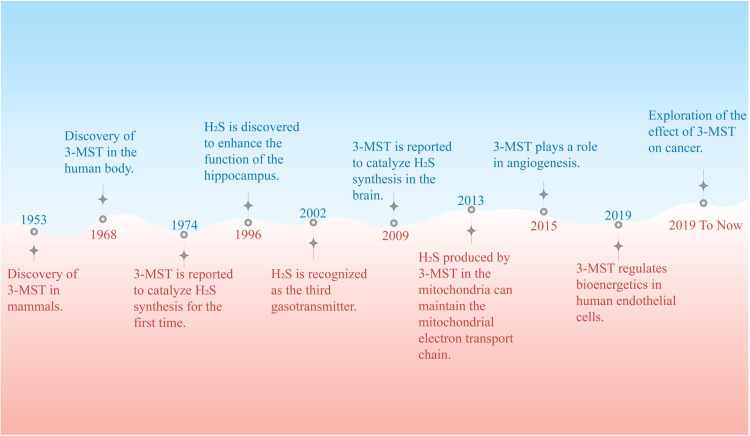
The discovery and research development of 3-MST. The diagram illustrates significant events in the study and development of 3-MST since its discovery in mammals. 3-MST, 3-mercaptopyruvate sulfurtransferase; H_2_S, hydrogen sulfide.

In 1953, during an investigation into the metabolism of cysteine (Cys), 3-MST was initially identified in tissues of mammals [11]. In 1968, researchers conducted a screening for cystinuria in patients at a charitable institution. During an examination of the urine of a patient with intellectual disability resulting from a sibling mating, researchers identified an unrecognized sulfur-containing metabolite known as 3-mercaptolactate [12]. Through their research, it is found that this patient lacked an enzyme that normally converts Cys-derived 3-MP into pyruvate. This missing enzyme is 3-MST. In the absence of 3-MST activity, 3-MP accumulates and is eventually converted to 3-mercaptolactate by lactate dehydrogenase [13]. In 1974, a study proposed that a cytoplasmic transaminase coupled with 3-MST could catalyze the production of H_2_S from Cys [14]. However, since the physiological role of H_2_S was not yet discovered at that time, this study did not attract widespread attention. 3-MST was only recognized as a cyanide antidote at that time. In 1996, studies demonstrated that physiological levels of H_2_S selectively potentiate N-methyl-d-aspartate receptor-mediated neuronal responses in the brain and facilitate the induction of long-term potentiation within the hippocampus [15]. This report challenged the previous notion of H_2_S as a toxic gas and shifted researchers' focus to the study of the physiological functions of H_2_S. By 2002, H_2_S was recognized as the third gasotransmitter after nitric oxide (NO) and carbon monoxide [16]. Subsequently, research into the biological effects of H_2_S has grown exponentially, and H_2_S synthesis is also extensively studied. In 2009, the ability of 3-MST was discovered to catalyze H_2_S synthesis from Cys in the brain, sparking widespread interest among researchers [17]. Since then, many physiological functions of 3-MST have been identified. An investigation revealed that H_2_S, synthesized by 3-MST within mitochondria, plays a crucial role in sustaining mitochondrial electron transport and underpinning cellular bioenergetic processes [18]. In 2015, researchers confirmed that activation of 3-MST by 3-MP could stimulate endothelial cell (EC) proliferation, migration, and angiogenesis in mice, suggesting that 3-MST may play a role in angiogenesis [19]. In 2020, it was reported that 3-MST modulated the bioenergetic and morphogenic properties of angiogenic functions in human ECs [20]. Recently, the role of 3-MST in cancer cells has also been explored (Fig. 1).

In the early stage, the research progress of 3-MST was limited due to the lack of effective or specific 3-MST inhibitors. In recent years, this situation has changed. The emergence of drug inhibitors of 3-MST opens a new round of pharmacological research on 3-MST and its role in health and disease. Three most commonly used 3-MST inhibitors include 2-[(4‑hydroxy-6-methylpyrimidin-2-yl)sulfanyl]-1-(naphthalen-1-yl)ethan-1-one (HMPSNE) [21,22], 2-ketobutyric acid [23], and aspartic acid [24], while aspartic acid inhibits the H_2_S production catalyzed by 3-MST indirectly by inhibiting cysteine aminotransferase (CAT). The discovery of colon cancer cell line CT26 also provides new momentum for in-depth studies on 3-MST due to the down-regulation (or lack) of expression of the other two endogenous H_2_S-catalyzing enzymes [25]. Mercaptolactate-cysteine disulfiduria (MCDU) is considered a hereditary metabolic disorder caused by congenital deficiency or absence of 3-MST [12,[26], [27], [28], [29]]. A research in 2013 first reported the use of MCDU mice as a model for 3-MST knockout mice in experiments [30]. This 3-MST knockout model has since been widely applied [3]. A recent study has reported the achievement of a stable lentiviral-mediated knockdown of 3-MST in human umbilical vein endothelial cells, employing two distinct short hairpin ribonucleic acid (shRNA) constructs [20]. Small interfering RNA (siRNA) is also widely used to silence 3-MST [31,32].

### The structure of 3-MST

3-MST is a 33 kDa protein belonging to rhodanese-like sulfur transferase family. It contains two Rhodans-like domains and exists in monomer-dimer equilibrium. The localization of the active site of 3-MST resides at the juncture of the two domains, whereas the majority of the residues crucial for binding and catalytic activity are situated within the C-terminal region. There is a catalytic site Cys247 at the C-terminus of 3-MST, which acts as a sensing switch for redox reactions within the subunit. 3-MST exists in a dynamic form of monomer dimer, with the active being the 3-MST monomer [[33], [34], [35], [36]]. In this process, the cleavage of the C-S bond in 3-MST is noteworthy, as 3-MST promotes the cleavage of the C-S bond without the participation of any cofactors. 3-MP, as a special thiol-containing metabolite, has an unstable sulfur [37]. When the thiolate moiety at the active site of 3-MST initiates an attack on the sulfur atom of 3-MP, the resulting cleavage of the C-S bond yields pyruvate enolate, which is anticipated to serve as an effective leaving group. Subsequent protonation of the enolate enhances its propensity as a leaving group, leading to its spontaneous decomposition into pyruvate and sulfite [38].

### The expression of 3-MST

3-MST is present in all tissues. Different species and tissues contain different levels of 3-MST. In animal experiments, 3-MST activity is detected in various tissues [39], with the highest activity found in the kidney and the next highest in the liver [40]. The 3-MST activity in the heart, lung, cerebellum, thymus, cerebrum, and testes is less than half that of the kidney. In guinea pigs [40], sheep [41], and cattle [41], anti-3-MST antibodies cannot be obtained by biochemical methods alone. In studies on rats, it has been found that in the liver, 3-MST is mainly distributed in the hepatocytes around the central vein, which is very active in drug metabolism [[42], [43], [44]]. In the kidney, 3-MST is mainly located in the epithelial cells of the proximal renal tubules [45]. In the myocardium, 3-MST is concentrated in the perinuclear area; in the lung, 3-MST is abundantly distributed in the ciliated and non-ciliated cells of the bronchiolar epithelium, but is less distributed in the alveolar area [46]. In the brain, 3-MST is more distributed in glial cells, but is not obviously observed in nerve cells. In the testes, a large amount of 3-MST is distributed in the interstitial cells. In the pancreas and small intestine, no significant distribution of 3-MST is observed. In 2012, Nagahara et al. found that 3-MST was also distributed in blood vessels [47]. 3-MST is also detected in the submandibular gland of mice. However, the expression of 3-MST in various organs of the body is mostly studied in animals as research subjects, and there is no systematic detection for humans. In pathological states, the expression level of 3-MST will change. For example, the level of 3-MST in the myocardium of heart failure patients is significantly reduced [48], and the expression of 3-MST is increased in human Down syndrome fibroblast cell models [49].

3-MST is commonly recognized as a 'mitochondrial enzyme' [46,50]. However, it is also found in the cytoplasm [14]. This may be related to the two splice variants with different localization of 3-MST: thiouridine modification protein 1 (TUM1)-Iso1 is located in the cytoplasm, while TUM1-Iso2 is present in both cytoplasm and mitochondria [51]. In rat liver, the specific activity of mitochondrial 3-MST is about twice that of cytoplasmic 3-MST [39,52]. The two additional H_2_S-synthesizing enzymes, CBS and CSE, are either sparsely distributed in mitochondria or transferred to mitochondria under certain circumstances, such as malignancies [[53], [54], [55]].

### The enzymology of 3-MST

**Fig. 2:**
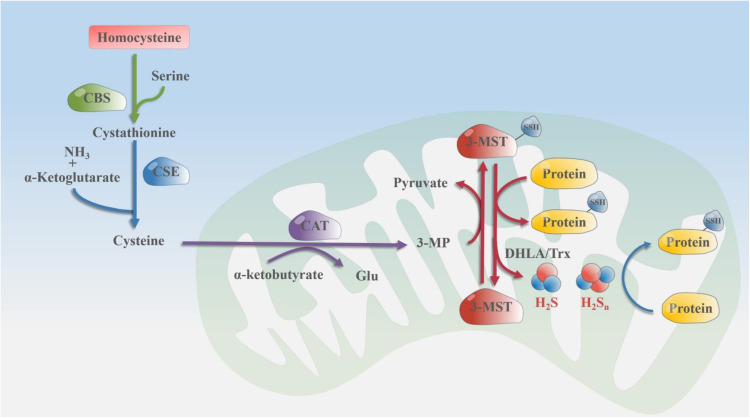
The enzymology of 3-MST. 3-MST plays an important role in redox regulation and cellular sulfur metabolism, and its role is mainly reflected in the production of endogenous H_2_S and polysulfide, and through H_2_S and polysulfide to produce protein oversulfur, in addition, 3-MST can also directly through the transfer of sulfur to overvulcanize proteins. The pathway begins with the conversion of homocysteine to cysteine (mediated by CBS), followed by the conversion of cysteine to cysteine by CSE. Entering the mitochondria, it participates in the production of H_2_S and polysulfide mediated by 3-MST and in the protein-based sulfur transfer pathway: The cysteine transferred to the mitochondria first passes through CAT to produce 3-MP, which is an important substrate for 3-MST to produce H_2_S. 3-MST reacts with 3-MP to form 3-MST intermediates, which pervulcanize proteins or are reduced to H_2_S and polysulfides catalyzed by DHLA/Trx. Moreover, since 3-MST is present in both the cytoplasm and mitochondria, the aforementioned pathway mediated by 3-MST also occurs in the cytoplasm (not described in detail in the figure). 3-MP: 3-mercaptopyruvate, 3-MST: 3-mercaptopyruvate sulfurtransferase, CAT: cysteine aminotransferase, CBS: cysteine beta-synthase, CSE: cysteine gamma-lyase, DHLA: dihydrolipoic acid, Glu: glutamate, H_2_S: hydrogen sulfide, H_2_S_n_: polysulfides, NH_3_: ammonia, Redox: oxidation/reduction, Trx: thioredoxin.

3-MST, along with CSE and CBS, is considered the enzymatic source of endogenous H_2_S in various cells and tissues [[56], [57], [58]], while a minor fraction of H_2_S is also generated through non-enzymatic pathways. However, unlike CSE and CBS, which use Cys and homocysteine as substrates [[59], [60], [61], [62]], 3-MST catalyzes the production of H_2_S using 3-MP as the sulfur donor [63,64]. During the process of H_2_S generation catalyzed by 3-MST, CAT and diamine oxidase (DAO) first generate 3-MP from L-Cys and D-Cys, respectively. CAT facilitates the transfer of an amino group from L-Cys to α-ketoglutarate, yielding l-glutamate and 3-MP, whereas DAO catalyzes the conversion of D-Cys. In the context of the presence of endogenous cofactors, namely thioredoxin (Trx) and dihydrolipoic acid (DHLA), 3-MST transfers sulfur from 3-MP to itself, forming a persulfide intermediate 3-MST-Cys-S-SH [62]. In the presence of reducing agents like Trx, the external sulfur atom (-S-SH) of the persulfide is transferred to protein or small molecule thiol receptors (reducing agents), leading to the persulfidation (RS-SH), and producing pyruvate and H_2_S [65]. During the entire chemical reaction, the conformation of 3-MST changes. When it is the reactant in the reaction, it remains in its active monomeric form; Upon engagement with 3-MP, Trx, and DHLA, the conformation of 3-MST is altered, resulting in the formation of a biologically inert dimeric state. The dimer is formed by the oxidation of exposed Cys residues and the generation of inter-subunit disulfide bonds [24]. The H_2_S production of the defective 3-MST mutant (the active site of the mutant Cys247 is replaced by serine) is 1.2 % of the H_2_S produced in the presence of wild-type 3-MST, indicating that Cys247 is the key site for 3-MST to produce H_2_S. After synthesis in different cell compartments (such as mitochondria), free forms of H_2_S can be released into the cytoplasm, or can be stored in the cell as a hydrogen polysulfides for subsequent release of H_2_S [66] (Fig. 2).

Additionally, 3-MST is an enzyme that not only generates H_2_S but also plays a role in the synthesis of intracellular polysulfides. An analysis of the brominated alkane adducts of polysulfides and H_2_S demonstrates that when exposed to 3-MP, the synthesis of H_2_S_3_ in cells of wild-type mice increased by roughly 65-fold compared to the control group, whereas H_2_S increased by around 5-fold. This suggests that 3-MST primarily functions in the synthesis of intracellular polysulfides [66].

## Expression of 3-MST in tumor tissues

3-MST expression is elevated in a wide range of tumors, such as human colon cancer [67], lung adenocarcinoma [68,69], bladder urothelial carcinoma [70,71], and oral squamous cell carcinoma [72].

In many tumor tissues, the expression levels of 3-MST are significantly higher than those of other H_2_S-producing enzymes. Glioblastoma cell line U-87 shows up-regulated 3-MST expression but downregulated CBS expression [66,73]. 3-MST has a higher expression level and activity than CSE in A375 and WM35 melanoma cell lines, SH-SY5Y neuroblastoma cell line, and U373 astrocyte cell line. Therefore, we can infer that 3-MST is a more important (than other endogenous H_2_S-producing enzymes) H_2_S source in these cells [73,74]. In many studies, the increase of 3-MST expression level is closely related to the deterioration of tumors and the low prognosis level. According to the widely accepted concept, the accumulation of successive mutations in colon epithelial cells transforms normal epithelial cells into epithelial polyps, followed by mutations that convert the cells into carcinoma in situ, ultimately leading to tumorigenesis [[75], [76], [77], [78]]. Another study indicates that during the successive mutations in colon epithelial cells, there is a gradual upregulation of 3-MST expression, accompanied by increased cell proliferation rates, oxidative phosphorylation, and even glycolysis, suggesting that 3-MST may mediate the carcinogenesis of colon epithelial cells by producing H_2_S and polysulfides [79]. OncoBase, a multi-type cancer somatic mutation database, describes the presence of 3-MST gene mutations in 36 cancers in the Cancer Genome Atlas [25]. Many *in vitro* studies show that hypoxia can promote the expression of 3-MST [80,81]. The expression of 3-MST increases as cancer cells recover from damage, such as stem cell-like transformation and multidrug resistance [82,83]. Therefore, the up-regulation of 3-MST is highly correlated with the stress resistance of cancer cells, indicating that cancer cells with increased 3-MST levels may better adapt to drug effects and their exuberant proliferation needs.

In contrast, 3-MST is moderately expressed in some tumor tissues such as surgical specimens of human papillary thyroid carcinoma, with no significant difference compared to surrounding tissues [84]. The enzyme activity of 3-MST in Ehrlich ascites cells derived from mouse liver is even lower than that in normal tissues [85]. For primary tumor tissues, the low expression level and low activity of 3-MST in human glioma are closely related to the increase of tumor malignancy [86]. Similarly, Li's team used Western blot to compare hepatocellular carcinoma (HCC) samples with paired non-tumor samples. The result showed that the transcription and expression of 3-MST in HCC samples was significantly decreased. Through clinical data analysis, it is found that the low expression of 3-MST is associated with tumor enlargement and poor survival [31]. These results seem to be inconsistent with the studies listed above. A reasonable explanation is that the functional roles of 3-MST in liver cancer and glioma differ from those in other cancer types. Although 3-MST exhibits higher expression in the liver compared to other tissues, CSE appears to be the primary enzyme responsible for H_2_S synthesis in the liver [87,88]. Genetic knockout of CSE reduces the production of the majority of H_2_S in the liver [89]. The H_2_S synthesis mediated by CSE requires appropriate regulatory mechanisms, and 3-MST seems to play a role in negative regulation. In HCC, downregulation of 3-MST stimulates H_2_S production, while overexpression of 3-MST significantly inhibits the formation of H_2_S [31]. This result may be due to the inhibitory effect of 3-MST on CSE; overexpression of 3-MST can suppress CSE, whereas downregulation of 3-MST can enhance the expression of CSE [90]. It is noteworthy that this is the first report of the negative regulatory effect of 3-MST on CSE, with the specific mechanism remaining unclear. Therefore, the primary intention in HCC may be to relieve the inhibition of CSE by downregulating 3-MST, promoting H_2_S production, and thus utilizing the cytoprotective function of H_2_S to protect itself from threats such as oxidative stress and apoptosis. Similar results have been observed in glioblastoma. Higher-grade glioblastoma exhibits lower expression of 3-MST, yet the levels of sulfane sulfur and the glutathione/oxidized glutathione ratio remain high, which may lead to elevated levels of H_2_S [86]. In summary, the reduction of 3-MST in HCC and neuroblastoma may share a common goal with the elevation of 3-MST in other cancer cells, that is, to increase H_2_S levels and promote cancer cell development. It is worth mentioning that some early studies compared the liver's expression of 3-MST activity or kidney homogenate with the activity of 3-MST in cancer cells, which shows that the expression level (or activity) of 3-MST in liver or kidney is the highest in all parenchymal organs [40]. Therefore, the 3-MST activity of cancer cells may be lower than that of these normal tissues, but they still have significant expression activity and an important functional role.

## The role of 3-MST in tumorigenesis and development

### 3-MST inhibits apoptosis of cancer cells

**Fig. 3:**
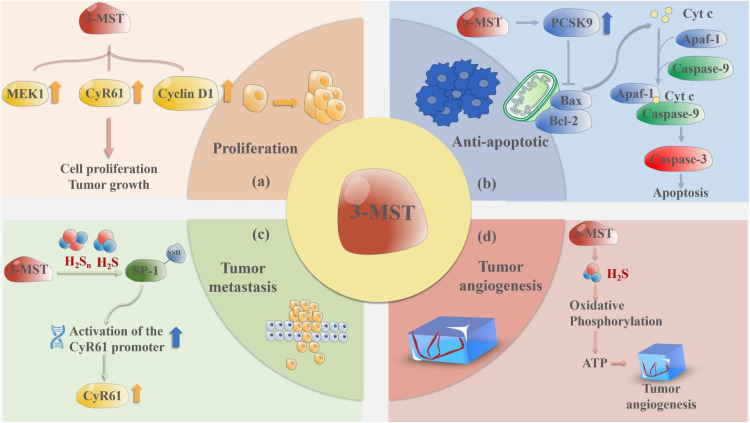
The role of 3-MST in tumors. The effects of 3-MST on various physiological and pathological pathways in the cell are diverse and extensive mainly by promoting tumor cell proliferation, inhibiting cell apoptosis, promoting tumor cell metastasis, promoting angiogenesis of tumor tissues and enhancing aerobic oxidation of cell mitochondria. (a) 3-MST increased MEK1, CyR61 and Cyclin D1 to promote cell proliferation and tumor growth; (b) Bax and Bcl-2 promote the transfer of cytochrome C from mitochondria to cytoplasm, form apoptotic complex with Apaf-1 and Caspase-9, activate Caspase-3 and promote apoptosis, while 3-MST can promote the expression of PCSK9. PCSK9 can inhibit the Bax/Bcl-2/Caspase-9/Caspase-3 pathway. (c) 3-MST oversulfates SP-1 with H_2_S and polysulfide, thereby activating the promoter of CyR61 gene, thereby increasing the CyR61 content; (d) 3-MST activates the NF-κB/IL-1β/VEGFR pathway and KATP/MAPK pathway through H_2_S. 3-MST can also inhibit the activity of cAMP and cGMP phosphodiesterase and increase the content of cAMP and cGMP, thus promoting the formation of tumor blood vessels. 3-MST: 3-mercaptopyruvate sulfurtransferase, Apaf-1: apoptotic protease activating factor 1, ATP: adenosine triphosphate, Bax: Bcl-2-associated X protein, Bcl-2: B-cell chronic lymphocytic leukemia/lymphoma-2, cAMP: cyclic adenosine monophosphate, cGMP: 3′,5′-cyclic guanosine monophosphate, CyR61: cysteine-rich angiogenic inducer 61, Cyt c: cytochrome c, H_2_S: hydrogen sulfide, H_2_S_n_: polysulfides, IL-1β: interleukin-1 beta, KATP: ATP-sensitive potassium, MAPK: mitogen-activated protein kinase, MEK1: mitogen-activated protein kinase kinases 1, NF-κB: nuclear factor-kappa B, PCSK9: proprotein convertase subtilisin/kexin type 9, SP-1: specificity protein-1, VEGFR: vascular endothelial growth factor receptor.

Apoptosis is the most common form of programmed cell death. Specifically, excessive apoptosis leads to tissue damage as well as many degenerative diseases. The apoptotic mechanism is complex and involves multiple pathways [91]. In the mitochondrial pathway, severe stress can inhibit B-cell leukemia/lymphoma-2 (Bcl-2)/Bcl-xL, or directly activate the pro-apoptotic member, Bcl-2-associated X protein (Bax)/Bcl-2 homologous antagonist killer (Bak). Then Bax/Bak acts on the mitochondrial outer membrane to form a protein pore, resulting in an elevation of permeability at the mitochondrial outer membrane. Subsequently, cytochrome C releases from the mitochondrial membrane space and combines with caspase-9 and apoptotic protease activator 1 to form the apoptotic body, which then continues to activate the caspase-3/7, thus leading to apoptosis [92]. The Janus kinase/signal transducer and activator of transcription signaling pathway serves as an upstream modulator of Bcl-2 expression, influencing apoptotic processes via the regulation of Bcl-2 function [[93], [94], [95]]. Numerous cancer treatments induce cell death by activating the apoptotic process and the related networks that govern cell demise. Proprotein convertase subtilisin/kexin type-9 (PCSK-9) is one of the key enzymes in the lipid transport process, which plays a vital role in the progression of various types of tumors. PCSK-9 can reduce the apoptosis of HCC by inhibiting the Bax/Bcl-2/Caspase-9/Caspase-3 pathway [96]. In the context of gliomas, PCSK-9 inhibition triggers the activation of caspase-3 and promotes apoptosis, while PCSK-9 overexpression suppresses apoptosis [97]. Therefore, reduction of PCSK-9 expression may promote apoptosis in cancer cells [98]. Katarina et al. showed that 3-MST silencing reduced PCSK-9 expression and promoted apoptosis, suggesting that 3-MST may exert anti-apoptotic effects by mediating PCSK-9 [98]. Furthermore, 3-MST silencing promoted the expression of tumor necrosis factor-like weak inducer of apoptosis, suggesting that the inhibitory apoptotic effect of 3-MST may be related to this substance. In conclusion, inhibition of 3-MST activity could exert anticancer effects by promoting apoptosis (Fig. 3).

Cell cycle arrest and apoptosis are closely related. Although direct evidence is still lacking to prove that it can induce apoptosis, cancer cells that have undergone cell cycle arrest are often more prone to apoptosis. Human cancer cells irradiated with blue light exhibit reversible cell cycle arrest and effectively induce apoptosis [99]. In colorectal cancer cells, betulin induces G0/G1 phase arrest and caspase-dependent apoptosis [100]. P53, a newly discovered tumor suppressor gene, is characterized by cell cycle arrest and induction of apoptosis [101]. The activator protein-1 (AP-1) transcription factor, which consists of the activating transcription factor Jun and the cyclic adenosine monophosphate (cAMP) response element-binding protein, can activate the promoter of cyclin D1, which is a key molecule in the G1-S phase transition [[102], [103], [104]]. The absence of cyclin D1 can induce cell cycle arrest. Studies have shown that HMPSNE significantly downregulates the levels of AP-1 and cyclin D1 in colon cancer cells, thereby triggering the cessation of the cell cycle and subsequent apoptosis [105]. These results indicate that 3-MST might participate in the regulation of the cell cycle, thereby suppressing apoptosis in cancer cells. The increase in calcium ion (Ca^2+^) concentration is involved in the early signal transduction of apoptosis and the execution phase of apoptosis. An increase in Ca^2+^ levels can result in the activation of mitochondrial permeability transition pores, Ca^2+^ overload in mitochondria, and mitochondrial swelling, ultimately causing the rupture of the mitochondrial outer membrane and triggering the mitochondrial pathway of apoptosis [106]. The silencing of the 3-MST gene induced by siRNA leads to an increase in Ca^2+^ concentration in colon cancer cells, accompanied by an increase in apoptosis, suggesting that 3-MST may be involved in the regulation of intracellular Ca^2+^, thereby inhibiting cancer apoptosis [98].

### The role of 3-MST in tumor metastasis

Metastasis is the cause of most cancer-related deaths, not the primary tumor [107]. The process of metastasis involves cancer cells detaching from the primary tumor, migrating and invading surrounding tissues, infiltrating nearby blood or lymphatic vessels, and then traversing the circulatory or lymphatic system to reach other parts of the body, where they establish new tumors [108]. Endogenous H_2_S produced by 3-MST significantly promotes cell proliferation and migration of murine breast cancer cells. Inhibition of H_2_S production through amino-oxyacetic acid (AOAA) treatment did not significantly affect the relocation of mouse mammary carcinoma cells. On the contrary, HMPSNE concentration-dependently inhibited it [109]. The specific mechanism is not yet clear.

Cysteine-rich angiogenic inducer 61 (CYR61) is a gene that has been identified to be associated with tumor migration and invasion. It can promote angiogenesis through signal transduction of αvβ3 receptors and downstream activation of AMP-activated protein kinase, protein kinase B, and the endothelial NO synthase/NO pathway system [110], thereby facilitating the migration and invasion of cancer cells. Epithelial-mesenchymal transition (EMT) involves the process of converting epithelial cells into mesenchymal cells constitutes a crucial stage in both embryonic development and tissue regeneration. However, it is utilized by cancer cells to perform a metastatic cascade, promoting their migration and invasion. Silencing CYR61 microRNAs inhibit the EMT process [111], accompanied by a reduction in migration and invasion, indicating that CYR61 can also promote cancer cell migration by promoting EMT [112]. 3-MST can promote the persulfidation of specificity protein 1 (SP1) by producing H_2_S and polysulfides, thereby activating the promoter of the CYR61 gene and increasing the level of CYR61 in colon cancer cells. The use of the 3-MST inhibitor HMPSNE can significantly inhibit the persulfidation of SP1 and reduce the expression of CYR61, suggesting that 3-MST may promote cancer cell migration and invasion by upregulating CYR61 [105,113,114] (Fig. 3).

Ras homolog gene family member A (RhoA) can facilitate the invasion and metastasis of cancer cells by regulating cell matrix adhesion and cytoskeleton reorganization [115,116], while the overexpression of sphingosine-1-phosphate (S1P) 2 leads to the activation of RhoA guanosine triphosphatase (GTPase), thus promoting cell migration [105]. Additionally, the overexpression of S1P2 can significantly increase the adhesion of glioma cells, which is an important aspect of cancer cell movement and invasion [[117], [118], [119]]. Therefore, S1P2 plays an important role in tumor metastasis. However, the application of the HMPSNE inhibitor targeting 3-MST, in contrast to the AOAA inhibitor of CBS, markedly suppressed the expression of messenger RNA and protein for sphingosine-1-phosphate receptor 2 (S1PR2) in colon cancer cells. These findings indicate that 3-MST may facilitate cell migration through the induction of S1PR2 expression [105]. Whether the mechanism by which 3-MST induces S1PR2 expression is related to protein persulfidation is not yet clear.

### 3-MST promotes tumor proliferation

The increased activity of 3-MST in colon cancer can promote the proliferation of cancer cells, while 3-MST specific inhibitor HMPSNE, but not AAOA, can significantly inhibit CT26 colon cancer proliferation [21,25]. Another study showed that application with 3-MST inhibitor HMPSNE could lead to a considerable drop in the messenger RNA levels of CYR61, which can inhibit cell growth and proliferation [105]. Simultaneously, the expression level of cyclin D1 exhibited a notable reduction, suggesting a halt in the progression of the cell cycle in cancer cells [105,[120], [121], [122]] (Fig. 3).

### 3-MST promotes tumor angiogenesis

Angiogenesis denotes the formation of novel blood vessels within the existing vascular network [123]. During the process of angiogenesis, previously quiescent ECs undergo activation and subsequently generate new blood vessels from the existing vasculature [124,125]. Angiogenesis is physiologically necessary for wound healing and tissue remodeling, but excessive angiogenesis promotes cancer [126]. In malignant tumors, angiogenesis can meet the demand for oxygen and nutrients required for the growth of the expanding tumor, promoting further proliferation of malignant cancer cells [127]. Moreover, tumors can secrete angiogenic factors (such as vascular endothelial growth factor (VEGF) to promote their growth and metastasis to distant organs, thus promoting their development [123]. For instance, following VEGF stimulation, VE-cadherin, which forms EC-EC junctions, undergoes internalization [128], consequently, vascular permeability is enhanced. The tumor vessels, characterized by their immature structure devoid of smooth muscle and pericytes, facilitate the transendothelial migration of cancer cells [129]. Metabolic changes in ECs play an important role in their activation process. Under physiological conditions, >85 % of cellular adenosine triphosphate (ATP) in ECs comes from glycolysis [130]. Mitochondria in ECs exhibit intact basal respiratory function and possess a high capacity for bioenergetic reserve [131], which may provide energy for angiogenesis in pathological states. Coletta et al.'s research showed that 3-MP can stimulate mouse EC proliferation, migration, and angiogenic sprouting by activating 3-MST [19]. Daily local administration of 0.3 mg/kg 3-MP significantly reduced the skin wound area in rats with full-thickness back burns. Since wound healing depends on the ability of angiogenesis, indicating that 3-MST can promote angiogenesis [19]. Inhibitors of 3-MST exert a dose-dependent suppressive effect on the proliferation of ECs and the process of angiogenesis, indicating that 3-MST holds a significant role in the angiogenic process [19] (Fig. 3). When 3-MST is silenced in ECs, there is a notable inhibition of mitochondrial oxidative phosphorylation and ATP synthesis. Concurrently, there is a significant accumulation of various glycolytic intermediates, such as fructose 1,6-bisphosphate, dihydroxyacetone phosphate, and 3-phosphoglycerate [19]. Lactate levels also tend to be higher, indicating increased glycolytic activity. Hyperglycemia can significantly inhibit the expression of 3-MST, and at the same time, mitochondrial function is significantly inhibited, and angiogenesis is also significantly reduced. Therefore, 3-MST may promote blood vessel generation by maintaining ATP synthesis in EC mitochondria. Inhibition of 3-MST may become an effective method to inhibit tumor angiogenesis [19].

### 3-MST improves tumor energy metabolism

Tumors need to obtain important nutrients from a barren environment (relative to normal cells) and use these nutrients to enhance their survival and proliferation [132]. When exposed to 3-MP, the substrate of 3-MST, the mitochondria will produce large amounts of H_2_S, which enhances mitochondrial activity and cellular bio-energy production, thus promoting the synthesis of ATP. There is decreased mitochondrial ATP production in 3-MST silenced cells [20], which may be related to the decreased efficiency of the tricarboxylic acid cycle and oxidative phosphorylation (with a slight promotion of glycolysis). The ability of 3-MP to stimulate mitochondrial electron flow and cellular bioenergy synthesis at 100–300 nM was lost in HCC cells with permanent attenuation of 3-MST or transiently silencing of 3-MST using siRNA [18]. Suppression of the activity of 3-MST could lead to a notable decrease in the basal rate of oxygen consumption and a partial, dose-dependent reduction in respiratory reserve capacity, indicating that 3-MST/3-MP is involved in the maintenance of resting bioenergy, which is generally dependent on endogenous H_2_S production in mitochondria [[133], [134], [135]].

## The effect of mitochondrial H_2_S produced by 3-MST

3-MST is detectable in both the cytosolic and mitochondrial compartments. However, the mitochondrial concentration of Cys is considerably higher than that in the cytoplasm [46,136], and the fact that the other two H_2_S-synthesizing enzymes, CBS and CSE, do not have physiological mitochondrial localization, the mitochondrial H_2_S is the most likely produced by 3-MST due to its location in mitochondria. Overall, we can attribute the effects of mitochondrial H_2_S and other polysulfides to 3-MST. The production of H_2_S and various polysulfides, facilitated by 3-MST, serves to sustain mitochondrial electron transport and bolster cellular bioenergetics [17,38,46]. Furthermore, it has been reported that the deletion of the 3-MST gene leads to a decrease in cellular antioxidant levels and an increase in basal reactive oxygen species (ROS) levels [48]. According to Li et al., the expression levels of 3-MST were decreased in myocardial samples from patients with severe heart failure at the end-stage, while CSE and CBS were unchanged, and mitochondrial H_2_S and polysulfides production were decreased, which correlated with elevated levels of oxidative stress, decreased mitochondrial mass, as well as modifications in structure and functionality [48]. In summary, these data suggest that 3-MST can maintain mitochondrial function by producing H_2_S and polysulfides. Additionally, mitochondrial H_2_S inhibits the activity of cAMP phosphodiesterase in mitochondria, resulting in an increase in mitochondrial cAMP levels, which could activate certain cAMP-dependent kinases (such as protein kinase A), thereby phosphorylating and activating a variety of key proteins in the mitochondrial electron transport chain and improving the efficiency of electron transport [[137], [138], [139], [140], [141]]. Therefore, the increased cAMP level in mitochondria induced by 3-MST-derived mitochondrial H_2_S may be beneficial for cancer cells to maximize the efficiency of electron transfer and ATP efficiency [142].

Mitochondrial fission is a tightly controlled process, and its disruption has the potential to modify metabolic pathways [143], proliferation [144], and apoptosis [145]. A crucial stage in the fission of mitochondrial membranes involves the recruitment of dynamin-related protein 1 (DRP1) to mitochondria, subsequently leading to membrane constriction facilitated by GTPase enzymatic activity [146]. Mitochondrial fission appears to maintain the redox homeostasis of cancer cells, thereby contributing to cancer cell survival and metastasis. Mitochondrial fission in potential brain metastatic cells can promote fatty acid oxidation, thereby maintaining cellular bioenergetics and redox homeostasis. Depletion of DRP1 leads to increased lipid droplet accumulation, impaired fatty acid oxidation, and weakened metastasis. Similarly, pharmacological inhibition of DRP1 reduces brain metastasis in breast cancer [147]. DRP1 oligomerization can protect HCC cells from ferroptosis. The absence of DRP1 oligomerization increases mitochondrial ROS accumulation and ferroptosis and inhibits tumor growth [148]. Knockdown of the 3-MST gene upregulates DRP1 levels, thus promoting mitochondrial fission and metastasis in colon cancer cells. Interestingly, 3-MST knockdown can not promote the survival of colon cancer cells but instead induce apoptosis [98]. Therefore, one possible explanation is that 3-MST knockdown can lead to mitochondrial metabolic abnormalities, decrease H_2_S production, and increase ROS, and colon cancer cells may maintain cellular redox homeostasis by upregulating DRP1 levels. However, this compensatory effect is not sufficient to save them. Additionally, an important purpose of mitochondrial fission is to separate damaged mitochondrial components and degrade them [149], and enhanced fission may mean that 3-MST knockdown leads to mitochondrial damage. In summary, 3-MST may affect DRP1-mediated mitochondrial fission by regulating the redox homeostasis of cancer cells.

## Limitations of existing researches on 3-MST

### Insufficient 3-MST related inhibitors

Over the past decades, only non-selective and weak substrate-like inhibitors have been reported. CAT catalyzes the transamination and reverse reactions between L-Cys and 2-ketoglutarate, thus producing 3-MP, the substrate for 3-MST. In 1982, L-aspartate was proven to be a potent inhibitor of the CAT reaction, competing with L-Cys for the binding site on the enzyme. Therefore, L-aspartate is considered an indirect 3-MST inhibitor by inhibiting CSE [150].

In 1995, 3-mercaptopicolinic acid (3-MPA) was identified as a non-competitive inhibitor of 3-MST, capable to associate with both the free enzyme and the enzyme-substrate conjugate, but with a significantly lower affinity for 3-MST than that of 3-MP. 2-MPA is also considered an inhibitor of 3-MST, but unlike 3-MPA, it has the capacity to exclusively interact with the enzyme-substrate complex, rather than with the free enzyme alone. Considering the difference between 2-MPA and 3-MPA is the position of the thiol group, therefore the position of the thiol group may be the main reason for the functional differences between 2-MPA and 3-MPA [151]. In 1996, researchers reported the inhibitory effects of three alpha-keto acids on 3-MST and identified them as inhibitors of 3-MST, including a-ketobutyrate, a-ketoglutarate, and pyruvate. However, these three inhibitors are all non-competitive. They do not compete for binding at the active site of 3-MST but bind to other sites, causing conformational changes in 3-MST, and leading to the observed inhibition [152]. The inhibitory effects of these non-selective or weak substrate-like inhibitors are not significant and are not suitable for biological research. Recently, researchers identified four new types of 3-MST inhibitors from a library of 174,118 compounds using high-throughput screening and HSip-1 fluorescence analysis [22]. From a pharmacological standpoint and with regard to potential translational applications, the compound known as HMPSNE stands out as the most intriguing among these, owing to its specific targeting of 3-MST. HMPSNE has indeed greatly advanced the study of 3-MST, but since then, no further attempts have been made to screen for more inhibitors. Nevertheless, HMPSNE has been found to not only inhibit 3-MST but also to downregulate the expression of both 3-MST and CBS [105]. More research needs to be conducted to find other selective 3-MST inhibitors or to modify HMPSNE by improving its selectivity.

### Insufficient mechanistic investigation of 3-MST in tumor

Currently, research on the role of 3-MST in cancer is mostly limited to observations of its inhibition or promotion. Nevertheless, the specific mechanisms of action of 3-MST in tumors are still unclear. Although there are some studies demonstrating part of the mechanisms, these theories still require extensive experimentation for validation. H_2_S and polysulfides are the main products of 3-MST catalysis. Experiments have shown that H_2_S and polysulfides play an important role in the development of tumor. H_2_S provides a potent immunological microenvironment for colon cancer [153] and increases the proliferation, invasion, and metastasis of bladder cancer [154]. The polysulfuration of Cys can inhibit the function of tumor suppressor factors [155]. However, the role of H_2_S and polysulfides in the promoting effect of 3-MST on tumors is still unknown, and the downstream signaling pathways have not yet been studied. Polysulfidation has a wide range of physiological effects. 3-MST is considered to be a major source of polysulfides *in vivo* [66]. Therefore, the biological effects of 3-MST have been greatly underestimated. In addition, unlike CBS and CSE, 3-MST is distributed in the cytoplasm and can also be localized in the mitochondria [36,50,51]. This special distribution may suggest that energy metabolism could be a target of 3-MST. However, there is still a gap in related studies. Research on the mechanism of action of 3-MST in cancer cells is still extremely insufficient, and any new evidence in this field will be pioneering.

## Discussion

As the conclusion of our review, we have illustrated the disturbances of 3-MST expression in an array of tumors, including human colon cancer, lung adenocarcinoma, bladder urothelial carcinoma, HCC, and so on. In recent years, 3-MST has shown excellent capabilities in tumorigenesis and development, the underlying mechanisms have also begun to diversify. Studies have found that 3-MST could inhibit the apoptosis of cancer cells, promote cancer metastasis, proliferation, angiogenesis, and improve the energy metabolism efficiency of cancer cells. However, there are still some urgent problems that need to be solved. For example, there is only one selective inhibitor of 3-MST, we need to identify other selective 3-MST inhibitors to provide more alternations for researchers. Additionally, the mechanism research is still insufficient, the participation of H_2_S or polysulfide is not included in the research scope. In the future, more studies on the role of 3-MST in cancer development and progression should be conducted and the underlying mechanisms will be illustrated more clearly.

## Declarations

Not applicable.

## Competing interest

The authors declare that they have no competing interests.

## Ethics approval and consent to participate

Not applicable.

## Consent for publication

Not applicable.

## Availability of data and material

Not applicable.

## CRediT authorship contribution statement

**Ka Zhang:** Writing – original draft. **Yi-Wen Zhu:** Conceptualization. **Ao-Qi Tang:** Data curation. **Ze-Tao Zhou:** Data curation. **Yi-Lun Yang:** Formal analysis. **Zi-Hui Liu:** Funding acquisition. **Yan Li:** Investigation. **Xiao-Yi Liang:** Conceptualization. **Zhi-Fen Feng:** Data curation. **Jun Wang:** Methodology. **Tong Jiang:** Software. **Qi-Ying Jiang:** Supervision. **Dong-Dong Wu:** Writing – review & editing.

## Declaration of competing interest

The authors declare that they have no known competing financial interests or personal relationships that could have appeared to influence the work reported in this paper.
